# 

**Published:** 1888-11

**Authors:** 


THE AMERICAN PUBLIC HEALTH ASSOCIATION will hold its sixteenth annual meeting at Milwaukee Tuesday, Wednesday, Thursday and Friday, November 20, 21, 22 and 23, 1888. The Executive Committee has selected the following topics for discussion at the meeting:

1. The Pollution of Water Supplies.
2. The Disposal of Refuse Matter of Cities.
3. Animal Diseases Dangerous to Man.
4. Maritime Quarantine, and Regulations for the Control of Contagious and Infectious Diseases, and their Mutual Relations.

Within the past few days, Dr. Oscar C. DeWolf, Health Commissioner of Chicago, has visited Buffalo for the purpose of examining into the present system employed in the disposal of garbage here, as he proposes to take part in the discussion of that subject at this meeting.

---

THE AMERICAN ACADEMY OF MEDICINE will hold its annual meeting in New York November 13 and 14, 1888.

### BOOKS AND PAMPHLETS RECEIVED.

Photographic Illustrations of Skin Diseases. Second series, Parts VII. and VIII. By Dr. George Henry Fox, New York. E. B. Treat, publisher. 1888. Price, $2.00 per part.

Hand-Book of Historical and Geographical Phthisiology, with special reference to the Distribution of Consumption in the United States. Compiled and arranged by Dr. George A. Evans, Brooklyn. D. Appleton & Co., publishers. Price, $2.00.

The Scientific Transactions of the Royal Dublin Society. December, 1887, April, 1888. The Scientific Proceedings of the Royal Dublin Society. July, November, 1887, February, May, 1888.

How Far can Legislation Aid in Maintaining a Proper Standard of Medical Education? By Mr. W. A. Purrington, Counsel of the Medical Society of the County of New York. 1888. Author's reprint.

Eine neue Anwendung der Paraffin-Methode. By Dr. Wm. C. Krauss (Attica, N. Y.) Berlin. 1888. Author's reprint.

Anatomischer Befund bei einer diptherischen Lähmung. Aus dem Laboratorium des Prof. Dr. Mendel in Berlin. Leipzig. 1888. Author's reprint.

On Some Novelties in my Electrical Treatment of Uterine Fibroids, with Answers to Objections. By Dr. G. Apostoli (Paris). Read at the Meeting of the British Medical Association, Glasgow. August, 1888. Author's reprint.

Papillomatous Cystic Tumor of Ovary, with a Hernial Pouch Developed in the Cicatrix of the Abdominal Wound from a Former Ovariotomy. By Dr. L. H. Laidley, St. Louis. 1888. Author's report.

Hot Water in the Management of Eye Diseases. Some Suggestions. By Dr. Leartus Connor, Detroit. 1887. Author's reprint.

The Preferable Climate for Phthisis; or, The Comparative Importance of Different Climatic Attributes in the Arrest of Pulmonary Diseases. By Dr. Charles Denison, Denver. 1888. Author's reprint.

Experimental Researches Respecting the Relation of Dress to Pelvic Diseases of Women. By Dr. J. H. Kellogg, Battle Creek, Mich. 1888. Author's reprint.

The Eye as a Factor in Functional Nervous Diseases. By Dr. F. Park Lewis, Buffalo. 1888. Author's reprint.

Displacements of the Uterus, together with Formula for Official Dilute Hydrobromic Acid of the United States Pharmacopeia. Dy Dr. DeWitt Clinton Wade, Holly, Mich. 1888. Author's reprint.

Some of the Advantages of the Union of Medical School and University. An Address delivered at Yale University June 26, 1888, by Dr. William H. Welch, Baltimore. Author's reprint.

A Case of Extra-Uterine Pregnancy; Embryo Destroyed by a Twenty-Milliampère Current without Interruptions; together with Remarks on the Best Manner of Using Electricity for this Purpose. By Dr. A. H. Buckmaster, Brooklyn. 1888. Author's reprint.

Is the Frequent Use of Forceps Abusive? By Dr. Thomas Opie, Baltimore. Read before the American Association of Obstetricians and Gynecologists in Washington, September 19, 1888. Author's reprint.

A Discussion of the General Principles Involved in the Operation of Removal of the Uterine Appendages. By Lawson Tait, F. R. C. S., Birmingham, Eng. 1886. Author's reprint.

The Treatment of Empyema: The Process of Repair. A Method of Subcutaneous Drainage and Irrigation, with Illustrative Cases. By G. J. Robertson, M. B. C. M., Surgeon to the Oldham Infirmary. 1888. Author's reprint.

The Significance of the Epiblastic Origin of the Central Nervous System. Presidential address before the New York Neurological Society, May 1, 1888. By Dr. George W. Jacoby, New York. 1888. Author's reprint.

The Causation of Cold Weather Diseases. By Dr. Henry B. Baker, Lansing, Mich. 1887. Author's reprint.

Health of Michigan in September, 1888. State Board of Health Report.

Weekly Abstracts of Sanitary Reports, U. S. Marine Hospital Service. Abstracts 40, 41, 42 and 43. October, 1888.

Report of Deaths and Contagious Diseases, Newport, R. I., for September, 1888.

---

### NEW BOOKS AND JOURNALS ANNOUNCED.

The Case of Emperor Frederick III. Full official report. By the German Physicians and Sir Morell Mackenzie. Cloth, $1.25; paper, 75 cents. Address the publisher, Edgar S. Werner, 48 University Place, New York.

The J. B. Lippincott Company, of Philadelphia, announces a new edition of the United States Dispensatory in preparation, and which is nearly ready for delivery.

The Western Publishing House, Chicago, announces that the Physician's Anatomical Aid, which has been in course of preparation for several years, is now about ready to issue.

A. L. Chatterton & Co., of New York, announce a new quarterly, *The Journal of Ophthalmology, Otology, and Laryngology*, to be issued in January, 1889. It will be edited by George S. Norton, M. D., assisted by Charles Deady, M. D. Subscription price, $3.00 a year.

Lindsay & Blakiston's Physician's Visiting List for 1889. P. Blakiston, Son & Co., publishers, 1012 Walnut street, Philadelphia. Prices, from $1.00 to $3.00.

